# Reviews

**Published:** 1880-02

**Authors:** 


### Reviews.

---

**The Theory and Practice of Medicine.** By Frederick T. Roberts, M. D., B. Sc., F. R. C. P. With Illustrations. Third American from the fourth London edition. Philadelphia: Lindsay & Blakiston. 1880.

The author of this very valuable contribution to the principles and practice of medicine has "endeavored to bring the information which it contains as nearly as possible up to the present date." While the numerous standard authorities, such as Aitkin, Watson, Bristowe, Flint and others, compassing almost the same field of medicine, would seem to leave but meagre encouragement for further effort, yet the fact that the present work has already passed to the fourth London edition and the third American edition shows how ably our author has performed the task, and how thoroughly his work is appreciated by the profession. A critical examination of the work unfolds the reasons for its growing popularity among students and practitioners, for whom it is written. Its conciseness is especially noticable, and yet the effort to condense is not made at the expense of clearness of description.

On all the subjects which have excited discussion of late in the profession, the author does not hesitate to express the most advanced and enlightened opinions. For instance, in diphtheritic croup, he asserts that "the only possible hope lies in the performance of tracheotomy or laryngotomy," an operation which is growing in favor, on account of the terrible mortality attending this complication of pharyngeal diphtheritis. So also in obstruction of the bowels from strictures, strangulation, intussusception, the operation for opening the abdomen with a view of removing the cause, he thinks is decidedly permissible, if the case is otherwise hopeless. Indeed, we grow in interest as we read and examine the soundness of Dr. Roberts, not only in the theory he enunciates, but also in the practice based upon the principles he aims to establish.

The work is a most valuablē one, and our readers should secure it for study and reference. L.

---

**A Biographical Dictionary of Contemporary American Physicians and Surgeons.** Edited by WILLIAM B. ATKINSON, M. D., permanent Secretary of the American Medical Association. Second Edition, enlarged and revised. Philadelphia: D. G. Binton, 1880.

The publisher in his notice, states that the work is intended to include *all* who have visibly and publicly contributed "to the advancement of medical science, in the United States, during the present generation," rather a broad scope for a modest volume of 750 pages.

It is to be supposed that great care and discrimination have been exercised, not only in choosing from among the many honored names which adorn the annals of the American profession, but in furnishing correct data as to professional attainments, etc. In looking over the list of physicians in this locality, whose labors and skill "have visibly and publicly contributed," to the purpose above indicated, we find that twelve have been thus saved from oblivion, through the extreme consideration of Dr. Atkinson, while others, of which our city may well be proud, are allowed to go down to the grave "unnoticed and unhonored."

We may well conclude that such a work as the publisher here presents to the profession, if executed upon the plan stated in the preface, is not only premature, but fails to give a correct exhibit of medical men, whose abilities and attainments command for them the leading positions in their respective localities. We doubt, therefore, whether it fulfills any good purpose, except to flatter the vanity of the "fortunate few." L.

---

**Atlas of Human Anatomy.** Containing 180 large plates, arranged according to Drs. Oesterreicher and Erdl from their original designs, and those of the greatest anatomists of modern times. With full and explanatory text by J. A. Jeancon, M. D. Cincinnati, Ohio: A. E. Wilde & Co.

We have received a sample copy of this work, and are very favorably impressed with it. It possesses one advantage, viz: that all the plates are of life-size. The study of anatomy is very much facilitated by works of this character. Without good drawings and plenty of dissecting material, it is impossible to study anatomy with any benefit, and as the material is scarce in our colleges, the importance of good drawings cannot be over-estimated. The plates are clear, truthful and correct, and we recommend them heartily to students, old and young. M.

**Diseases of Women.** By LAWSON TAIT, F. R. C. S. Second edition, thoroughly revised and enlarged. Specially prepared for "Wood's Library," New York. Wm. Wood & Co., 1879.

The author's object in this book, is stated to be, to offer in a condensed form the results of his own experience, which is known to be very extensive, in the department of gynecology. The publishers have conferred a favor upon the American profession in placing so valuable a work within their reach.

The author's effort to be brief, robs many chapters of the clearness which is found in the larger treatises, such as Emmett and Barnes. Nevertheless, a vast amount of practical knowledge upon a most important class of diseases, is furnished in this modest little volume, valuable to the experienced gynecologist, in the practical hints it furnishes on many difficult subjects, and also to the younger practitioners, in giving a safe guide, on account of its marked conservatism, in the treatment of this class of diseases. The chapter on the ovaries, the most extensive in this work, will especially repay a careful perusal and study. The book maintains the high reputation of this series. L.

---

**Photographic Illustrations of Skin Diseases.** By GEORGE HENRY FOX, M. D., Professor of Dermatology, in Starling Medical College. New York: E. B. Treat, 805 Broadway.

The second, third and fourth parts of this excellent work have reached us. The second part contains four plates, Keloid, Rosasea, Psoriasis nummulata and Ichthyosis simplex. The third part contains five plates, Fibroma pendulum, Varicella, Zoster pertoralis and lumbalis, Eczema universale. The fourth part contains Leucoderma, Chromophytosis, Favus capitis and corporis, and Eczema cruris. The plates are all as life-like and truthful as possible. We consider them superior to anything yet published, and cannot be surpassed. M.

**American Cyclopœdia of Domestic Medicine and Household Surgery.** By SAMUAL PAYNE FORD, M. D. Chicago: E. P. Kingsley & Co. J. Hugill, Cincinnati. W. A. Edwards, St. Louis. 1879.

This is a large work of over twelve hundred pages. With a view to ready reference, the contents are arranged alphabetically. Unlike nearly all books with similar titles and purposes, it has not the slightest tinge of quackery; on the contrary, its author is a regular graduate of the Medical College of this city, as well as of the University of Toronto, Ontario, while the teachings and advice are sensible and sound throughout. All forms of popular superstition and quackery are discountenanced, and it has evidently been the author's aim to enlighten the ignorance of all classes. For this reason, we consider the introduction of this work opportune, and believe it the duty of physicians generally to recommend it to families in their care. V. P.

---

**Paracentesis of the Pericardium. A Consideration of the Surgical Treatment of Pericardial Effusions.** By JOHN B. ROBBERTS, M. D., Lecturer of Anatomy in the Philadelphia School of Anatomy. Philadelphia: J. B. Lippincott & Co.

The author has, with great diligence and care, gathered from journals and text-books 60 cases, in which this operation has been performed; 24 recovered and 36 died, the mortality being 60 per cent; since 1860 the operation has been performed 35 times, with 10 recoveries, the mortality for that period being 71-42 per cent, but the author calls attention to the fact, that there were serious and fatal complications in many cases. By excluding these cases, he finds, in 13 uncomplicated cases of pericardial effusion, that the result was ten recoveries and three deaths, the mortality being 23 per cent, a very favorable result, indeed. The author treats in different chapters of the etiology, symptoms, diagnosis, prognosis and treatment. The work is a well written and interesting little monograph. M.

**Memorial Oration in Honor of Ephraim McDowell, "The Father of Ovariotomy."** By SAMUEL D. GROSS, M. D.

There is something quite touching in this tribute of respect from one of the ablest to one of the oldest surgeons of this country. Not only are the claims of priority in ovariotomy fairly discussed, but a historical sketch of the operation is given, showing how much America has contributed to our knowledge in this department.

A well executed engraving of Dr. McDowell serves as the frontispiece, while the arrangement and typographical dress of the book reflect considerable credit upon the publishers. H.

---

**A System of Midwifery.** By WILLIAM LEISHMAN, M. D., Regius Professor of Midwifery, in the University, of Glasgow. Third American edition, revised by the author, with additions by John S. Parry, M. D. Philadelphia: Henry C. Lea. 1879.

The former editions of this work are well known in this country, and are universally praised. The death of Dr. John S. Parry, has thrown the work of American editor into other hands. Dr. Parry's former additions have been retained, and in the words of the author, in the present edition, "such alterations have been made, as the progress of obstetrical science seems to require."

The present edition is a work of something over seven hundred pages, and contains two hundred and five illustrations.

We can heartily recommend it as alike valuable to students and practitioners. V. P.

